# Decrease in CD4 and CD8 lymphocytes are predictors of severe clinical picture and unfavorable outcome of the disease in patients with COVID-19

**DOI:** 10.1515/med-2025-1246

**Published:** 2025-07-24

**Authors:** Biljana Popovska Jovičić, Ivana Raković, Nemanja Đorđević, Jagoda Gavrilović, Predrag Čanović, Ružica Radojević Marjanović, Sara Petrović, Katarina B. Milosavljević, Ana Divjak, Nenad Stanković, Milan Paunović, Miloš Z. Milosavljević

**Affiliations:** Department of Infectious Diseases, Faculty of Medical Sciences, University of Kragujevac, Kragujevac, 34000, Serbia; Clinic for Infectious Diseases, University Clinical Center Kragujevac, Kragujevac, 34000, Serbia; Institute of Emergency Medicine, Kragujevac, 34000, Serbia; Department of Physical Medicine and Rehabilitation, Faculty of Medical Sciences, University of Kragujevac, Kragujevac, 34000, Serbia; Department of Physical Medicine and Rehabilitation, University Clinical Center Kragujevac, Kragujevac, 34000, Serbia; Clinic for General Surgery, University Clinical Center Kragujevac, Kragujevac, 34000, Serbia; Center for Pediatric Surgery, University Clinical Center Kragujevac, Kragujevac, 34000, Serbia; Department of Pathology, University Clinical Center Kragujevac, Kragujevac, 34000, Serbia; Department of Infectious Diseases, Faculty of Medical Sciences, University of Kragujevac, Svetozara Markovića 69, Kragujevac, 34000, Serbia; Clinic for Infectious Diseases, University Clinical Center Kragujevac, Zmaj Jovina 30, Kragujevac, 34000, Serbia; Faculty of Medical Sciences, University of Kragujevac, Kragujevac, 34000, Serbia

**Keywords:** CD4-positive T-lymphocytes, CD8-positive T-lymphocytes, severity of illness, fatal outcome, COVID-19

## Abstract

**Study objective:**

The main objective of this study was to investigate the association between CD4+ and CD8+ lymphocyte values and disease severity, need for oxygen therapy and disease outcomes.

**Design:**

The research was designed as a cross-sectional observational study.

**Setting:**

This study was conducted at the Clinic for Infectious Diseases, University Clinical Center Kragujevac, as a COVID-19 treatment center.

**Participants:**

The study group consisted of a total of 101 adult hospitalized patients with confirmed COVID-19 infection, excluding patients under 18 years of age, patients with malignant diseases, tuberculosis, hepatitis, immune disorders, pregnant women, or HIV-positive patients. SARS-CoV2 infection was diagnosed by rapid antigen tests or real-time reverse transcription polymerase chain reaction from a nasal swab.

**Interventions:**

The patients were classified into two groups based on oxygen therapy needs, disease severity, and disease outcomes.

**Results and conclusions:**

Low CD4+ and CD8+ T cell values were associated with severe clinical presentation, more need for oxygen therapy as well as poor disease outcome. Receiver operating characteristic analysis provided cutoff values to support predicting the aforementioned variables, establishing CD4+ and CD8+ values as significant prognostic biomarkers. Future studies should be aimed at identifying factors that lead to gender differences in the immune response.

## 1 Introduction

The twenty-first century has been defined by outbreaks of pandemics, presenting significant challenges to health, economies, and societies worldwide. COVID-19 has left an indelible mark on the globe, underscoring the imperative for readiness, decisive action, and international collaboration in tackling infectious diseases. Moving forward, the insights gleaned from these outbreaks will prove indispensable in lessening the impact of future public health emergencies. Identified as a novel strain of β-coronavirus, SARS-CoV-2 precipitated the rapid spread of COVID-19 [1], characterized by a spectrum of symptoms from mild respiratory issues to severe complications such as acute respiratory distress syndrome and multi-organ dysfunction [2].

The advent of SARS-CoV-2 and the ensuing COVID-19 pandemic necessitated swift advancements in comprehending the disease’s clinical presentation, transmission dynamics, and risk determinants. Timely identification of individuals at heightened risk of severe illness, based on factors such as underlying health conditions and diagnostic indicators, remains pivotal for optimizing clinical management and enhancing patient outcomes [3–5]. Sustained research efforts and data analysis are paramount to effectively address this evolving global health crisis.

Examination of demographic factors has revealed notable gender disparities in the prognosis and therapeutic requirements of COVID-19. Research indicates a higher susceptibility among males to severe outcomes, including increased mortality rates, necessitating oxygen therapy, and mechanical ventilation [6]. These distinctions stem from a complex interplay of biological variables, encompassing differences in immune responses and hormonal profiles, alongside lifestyle factors such as smoking prevalence and pre-existing health conditions [7]. Acknowledging these gender-specific nuances is pivotal, given the multifaceted nature of immune responses to COVID-19.

Immune responses to COVID-19 entail a dynamic interaction between innate and adaptive immunity mechanisms. The innate immune system acts as the body’s initial defense against SARS-CoV-2, while adaptive immunity confers long-term protection through humoral and cellular components, including CD4+ helper T cells and CD8+ cytotoxic T lymphocytes [8]. These immune cells, activated by antigen-presenting cells, play crucial roles in eradicating virus-infected cells and establishing immunological memory, facilitating a robust response upon re-exposure [9].

Numerous publications suggest that evaluating CD4+ and CD8+ T cell counts may provide significant prognostic information regarding the severity of COVID-19 [10], including mortality, admission to intensive care units, and recovery. In one prospective study involving 179 patients with COVID-19 pneumonia, a low CD8+ T cell count was a significant predictor of high mortality among patients of similar age and comorbidities [11–13]. Other studies suggest that a low CD4+ T cell count (<200 μL/L) predicts radiographic progression in severely and critically ill COVID-19 patients [14]. These findings indicate that the absolute counts of CD4+ and CD8+ T cells may be significant biomarkers of disease severity and recovery in COVID-19 patients [15].

There is a strong need for further research to explore the connection between CD4+ and CD8+ levels, as well as the CD4+/CD8+ lymphocyte ratio, with disease severity and outcomes in COVID-19. Future studies should aim to investigate the benefits of measuring lymphocyte subtype levels as prognostic biomarkers for disease severity, mortality, and response to treatment in patients infected with the SARS-CoV-2 virus. Comprehensive understanding of these immune mechanisms is crucial for devising effective strategies to manage and prevent COVID-19, thereby enhancing patient outcomes and mitigating the pandemic’s impact.

The aim of this study is to determine whether there is a statistically significant relationship between CD4+ and CD8+ lymphocyte counts and their relationship to clinical and demographic data, severity of clinical presentation, use of oxygen therapy, radiographic lung parameters (chest X-rays [CXR] score), and disease progression. Through receiver operating characteristic (ROC) analysis of CD4+ and CD8+ lymphocyte levels and the CD4+/CD8+ ratio, we also aim to establish cut-off values for lymphocytes as predictive variables for severity of clinical presentation, disease progression, and the need for oxygen therapy.

## 2 Materials and methods

### 2.1 Study design

The study was designed as an observational, cross-sectional study and included 101 patients with confirmed COVID-19 infection, diagnosed using a rapid antigen test or real-time reverse transcription polymerase chain reaction. Patients under 18 years of age, pregnant women, individuals with malignancies, hepatitis virus infections, tuberculosis, and those with immune system disorders or HIV were excluded from the study. This approach allowed for a more precise examination of a specific population of adults with confirmed COVID-19 infection, reducing the influence of factors not directly related to the study’s objective that could potentially confound the results.

### 2.2 Study groups

All patients in the study were classified based on their need for oxygen therapy. According to lung radiography and disease outcomes, they were divided into two groups.

Group with mild clinical presentation: this group included 36 patients who did not require oxygen therapy (with oxygen saturation ≥94%). CXR were described as normal or with pronounced interstitial infiltrates.

Group with severe clinical presentation: this group included 65 patients who required oxygen therapy (with oxygen saturation <94%). CXR were described as focal or multifocal consolidations of pulmonary parenchyma.

This division allowed researchers to analyze differences between patients with mild and severe clinical presentations, focusing on the need for oxygen therapy and the characteristics of lung radiographic findings. Depending on the severity of the clinical presentation, patients received different types of oxygen therapies, including standard oxygen, high-flow therapy, non-invasive ventilation, and mechanical ventilation. This differentiation allowed for the adjustment of therapy based on the severity of patients’ conditions.

For the analysis of lung radiographs, a model from the study conducted by Borghesi et al. in 2020 was used. According to the methodology of this study, chest X-rays were divided into six zones by two lines. Each of the six zones was assessed for lung changes, with scores assigned as follows: 0 – no changes, 1 – interstitial infiltrates, 2 – alveolar infiltrates, 3 – interstitial and alveolar infiltrates. Summing these scores in each lung zone provided a total score ranging from 0 to 18 (CXR) [16]. Based on this score, radiographic findings were classified as normal (CXR 0), diffusely pronounced interstitial infiltrates (CXR 1–6), focal consolidation of the pulmonary parenchyma (CXR 7–12), and multifocal consolidation (CXR 13–18). This classification allowed for precise analysis of changes in chest X-rays concerning the severity of the disease.

Blood samples from COVID-19-positive patients were taken via venipuncture immediately upon admission for a complete blood count, coagulation status (international normalized ratio, prothrombin time, and fibrinogen), biochemical analyses (C-reactive protein [CRP], procalcitonin, glucose, aspartate aminotransferase, alanine aminotransferase, creatine kinase MB, lactate dehydrogenase, pro-B type natriuretic peptide, troponin, ferritin, and potassium).

All laboratory analyses were conducted in the central laboratory of the University Clinical Center Kragujevac using standard methods on the Beckman Coulter AU 400 Unicel DXC 800 Synchron Clinical System.

Body mass index (BMI) was used to measure body mass based on weight and height. BMI was calculated as follows [17]:

\[\text{BMI}=\text{weight}\hspace{.25em}\text{(kg)/height}\hspace{.25em}{\text{(m}}^{2}\text{)}\text{.}]\]

Blood samples from COVID-19-positive patients were sent immediately upon admission to the Virology Laboratory at the University Clinical Center Kragujevac to calculate the absolute number of lymphocytes. We measured CD4+ and CD8+ lymphocyte counts, as well as the CD4+/CD8+ lymphocyte ratio, using the FACSCount system (DB Bioscience, San Jose, CA, USA). This system features an automated flow cytometer and reagent kits (BD FACSCount™ Reagent Kit, Sigma Aldrich) (reagents’ concentration − CD4+ PE 0.075 μg/mL; CD3 PE-Cy5 0.625 μg/mL, beads 1.29 × 10^5^ beads/mL; CD8+ PE 0.312 μg/mL, CD3+ PE-Cy5 0.625 μg/mL, beads 2.58 × 10^5^ beads/mL). The FACSCount system employs a two-tube, two-color immunofluorescence method with unlysed whole blood. Blood samples are mixed with pre-aliquoted reagent tubes containing fluorochrome-labeled antibodies that target CD3+, CD4+, and CD8+ lymphocyte surface antigens. After fixation, cells are analyzed on the FACSCount instrument, where they are exposed to a laser. This process generates light scatter and fluorescence data, which are used to count and analyze the cells. In addition to monoclonal antibodies, the reagent tubes contain fluorochrome-labeled reference beads that serve both as a fluorescent standard for locating lymphocytes and as a quantitation standard.

The FACSCount’s internal software (version 1.5) analyzes these controls and calculates absolute cell counts for CD3+, CD4+, and CD8+, as well as the percentages of CD4+ and CD8+ cells relative to total CD3+ cells and the CD4+/CD8+ ratio. The entire process is automated, with results printed directly by the instrument [18].

### 2.3 Ethical considerations

This study was conducted at the University Clinical Center Kragujevac, which was also a COVID center. Prior to the start of the research, the Ethics Committee of the University Clinical Center Kragujevac had approved the study (approval number/decision 01/20-498). All participants in the study signed informed consent. All research procedures were conducted in accordance with the Declaration of Helsinki and the Principles of Good Practice. This approach ensures ethical conduct, transparency, and accountability in carrying out the research, respecting the rights and well-being of the participants. The ethical framework and procedures aligned with these guidelines contribute to the integrity and validity of the study results, providing a basis for the reliability and relevance of the collected data.

### 2.4 Statistical analysis

Statistical analysis was conducted using the SPSS software package, version 26 (SPSS Inc., Chicago, IL, USA). The Mann–Whitney *U*-test compared mean values between two groups, while the Wilcoxon signed-rank test assessed laboratory parameter differences under various conditions. Spearman correlation coefficients were used to analyze relationships between two continuous variables. Fisher’s exact test evaluated 2 × 2 contingency tables. Multiple linear logistic regression was performed to predict CD4+ and CD8+ lymphocyte values.

The reliability of CD4+, CD8+ and the CD4+/CD8+ ratio as predictors for clinical outcomes and oxygen therapy needs was assessed through ROC curve analysis, which also determined cut-off values, sensitivity, specificity, and the area under the receiver operating characteristic curve (AUROC). Statistical significance was set at *p* < 0.05, with group comparisons presented as median (1Q, 3Q).


**Informed consent:** Informed consent has been obtained from all individuals included in this study.
**Ethical approval:** The research related to human use has complied with all the relevant national regulations, institutional policies, in accordance with the tenets of the Helsinki declaration, and has been approved by the authors’ institutional review board or equivalent committee.

## 3 Results

### 3.1 Decreased values of CD4+ and CD8+ lymphocytes in patients with COVID-19, in combination with relatively unchanged CD4+/CD8+ ratio, indicate a more severe clinical picture, need for oxygen therapy, and a fatal outcome

In the present study, we investigated 101 patients with COVID-19. Variations in CD4+ and CD8+ lymphocyte values, as well as the CD4+/CD8+ ratio were examined concerning the severity of the clinical presentation, disease outcome, and the use of oxygen therapy. The clinical characteristics of the patients involved in the study are presented in Table 1. Male gender was associated with a more severe clinical presentation, need for oxygen therapy, and fatal outcome. Comorbidities, especially the presence of arterial hypertension and diabetes mellitus, proved to be a risk factor.

**Table 1:** Demographic and clinical characteristics of the study population

Variables		Clinical features		Disease outcome		Oxygen therapy		*N*
		Mild	Severe	Survivors	Non-survivors	No	Yes	
Gender	Males	22/68 (32.4%)	46/68 (67.6%)	61 (89.7%)	7 (10.3%)	21 (30.9%)	47 (69.1%)	101
	Females	16/33 (48.5%)	17/33 (51.5%)	30 (90.9%)	3 (9.1%)	15 (45.5%)	18 (54.5%)	
Age Mdn (1Q, 3Q)		46 (37.5, 62)	65 (57, 71)	61 (42, 68)	68 (59.7, 77)	45 (36.5, 61.8)	63 (57, 70.5)	101
BMI Mdn (1Q, 3Q)		26 (23.6, 29)	28 (26.1, 31.1)	27.5 (25.3, 30.9)	27.7 (26.2, 29.9)	26 (23.8, 28.7)	28 (26.1, 31.1)	101
Comorbidities	No	23/41 (56.1%)	18/41 (43.9%)	38/41 (92.7%)	3/41 (7.3%)	21/41 (51.2%)	20/41 (48.8%)	101
	Yes	15/60 (25%)	45/60 (75%)	53/60 (88.3%)	7/60 (11.7%)	15/60 (25%)	45/60 (75%)	
Diabetes mellitus	No	35/84 (41.7%)	49/84 (53.5%)	76/84 (90.5%)	8/84 (9.5%)	33/84 (39.3%)	51/84 (60.7%)	101
	Yes	3/17 (17.6%)	14/17 (82.4%)	15/17 (88.2%)	2/17 (11.8%)	3/17 (17.6%)	14/17 (82.4%)	
Arterial hypertension	No	29/54 (53.7%)	25/54 (46.3%)	50/54 (92.6%)	4/54 (7.4%)	27/54 (50%)	27/54 (50%)	101
	Yes	9/47 (19.1%)	38/47 (80.9%)	41/47 (87.2%)	6/47 (12.8%)	9/47 (19.1%)	38/47 (80.9%)	

Data were obtained from the medical history (*n* = 101) and blood samples were taken straight after hospital admission. Mdn – median.

**Figure 1:**
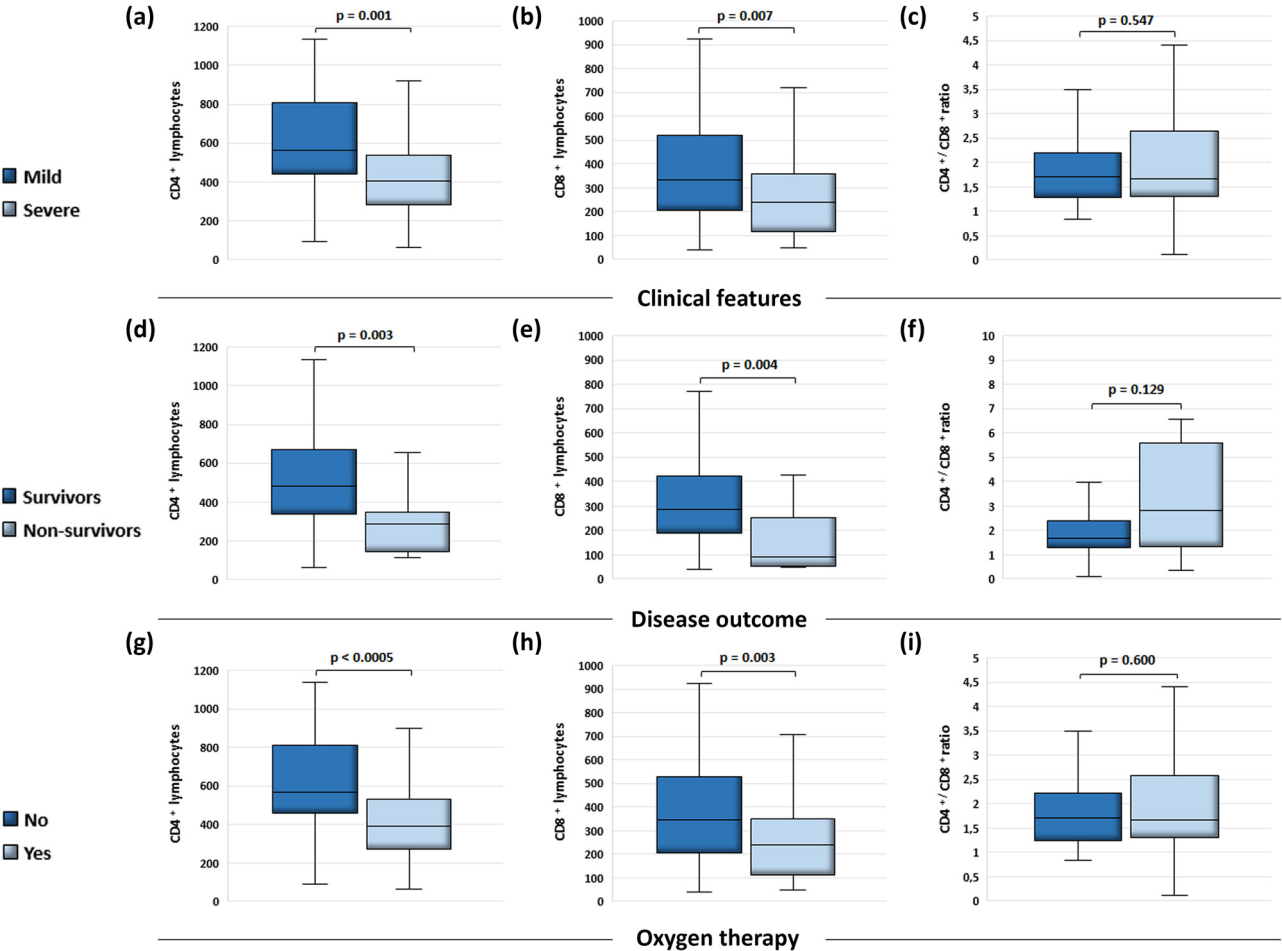
Blood values of CD4+, CD8+ lymphocytes and CD4+/CD8+ ratio, in COVID-19-positive patients and their relationship with the severity of clinical picture, the outcome of the disease, and the use of oxygen therapy. According to the disease severity the patients were divided into groups – mild (*n* = 38) and severe (*n* = 63) while they were classified into groups with favorable (*n* = 91) and fatal (*n* = 10) outcome according to the disease outcome. Considering the need for oxygen therapy, the patients were divided into a group that was treated with oxygen (*n* = 65) and the other that was not (*n* = 36). Data were obtained from the medical history (*n* = 101) and blood samples were taken straight after hospital admission. (a) Differences in CD4+ lymphocyte values (Mdn = 564 cells/mm^3^ (442.75, 807.75), *n* = 38 vs Mdn = 405 cells/mm^3^ (282, 537), *n* = 63; *p* = 0.001); (b) CD8+ lymphocyte values (Mdn = 332.5 cells/mm^3^ (204, 521.25), *n* = 38 vs Mdn = 239 cells/mm^3^ (116, 357), *n* = 63; *p* = 0.007); and (c) CD4+/CD8+ ratio (Mdn = 1.715 (1.2825, 2.2), *n* = 38 vs Mdn = 1.67 (1.3, 2.65), *n* = 63; *p* = 0.547) in patients with mild and severe clinical pictures. (d) Differences in CD4+ lymphocyte values (Mdn = 480 cells/mm^3^ (338, 669), *n* = 91 vs Mdn = 289 cells/mm^3^ (145, 350.75), *n* = 10; *p* = 0.003); (e) CD8+ lymphocyte values (Mdn = 288 cells/mm^3^ (189, 421), *n* = 91 vs Mdn = 90.5 cells/mm^3^ (51.75, 250.75), *n* = 10; *p* = 0.004); and (f) CD4+/CD8+ ratio (Mdn = 1.66 (1.3, 2.38), *n* = 91 vs Mdn = 2.83 (1.32, 5.58), *n* = 10; *p* = 0.129) in patients with favorable and fatal disease outcomes. (g) Differences in CD4+ lymphocyte values (Mdn = 568.5 cells/mm^3^ (461, 811.25), *n* = 36 vs Mdn = 390 cells/mm^3^ (274, 530), *n* = 65; *p* < 0.0005); (h) CD8+ lymphocyte values (Mdn = 346.5 cells/mm^3^ (207.5, 527.75), *n* = 36 vs Mdn = 239 cells/mm^3^ (113.5, 351), *n* = 65; *p* = 0.003); and (i) CD4+/CD8+ ratio (Mdn = 1.715 (1.2475, 2.22), *n* = 36 vs Mdn = 1.67 (1.315, 2.59), *n* = 65; *p* = 0.600) in patients with or without oxygen therapy. The Mann–Whitney *U*-test was used to determine statistical significance. Results were depicted as median (1Q, 3Q). Values of *p* < 0.05 were taken as statistically significant.

We observed lower blood values of CD4+ and CD8+ lymphocytes (Figure 1a and b), while the CD4+/CD8+ ratio remained relatively similar (Figure 1c) in the group of patients with a proved severe clinical presentation of COVID-19. Similarly, the patients with fatal outcomes of the disease exhibited lower values of CD4+ and CD8+ lymphocytes (Figure 1d and e). Remarkably, the CD4+/CD8+ ratio was found to be higher in the group of non-survivors compared to the survivors (Figure 1f), although the difference was not statistically significant (*p* > 0.05). Furthermore, in the patients requiring oxygen therapy, we observed lower blood levels of CD4+ and CD8+ lymphocytes (Figure 1g and h), while the CD4+/CD8+ ratio remained relatively unchanged (Figure 1i).

### 3.2 A decline in the values of CD4+ and CD8+ lymphocytes, accompanied by a marginal concurrent rise in the CD4+/CD8+ ratio, resulted in an elevation of the radiographic score among individuals afflicted by COVID-19

**Figure 2:**
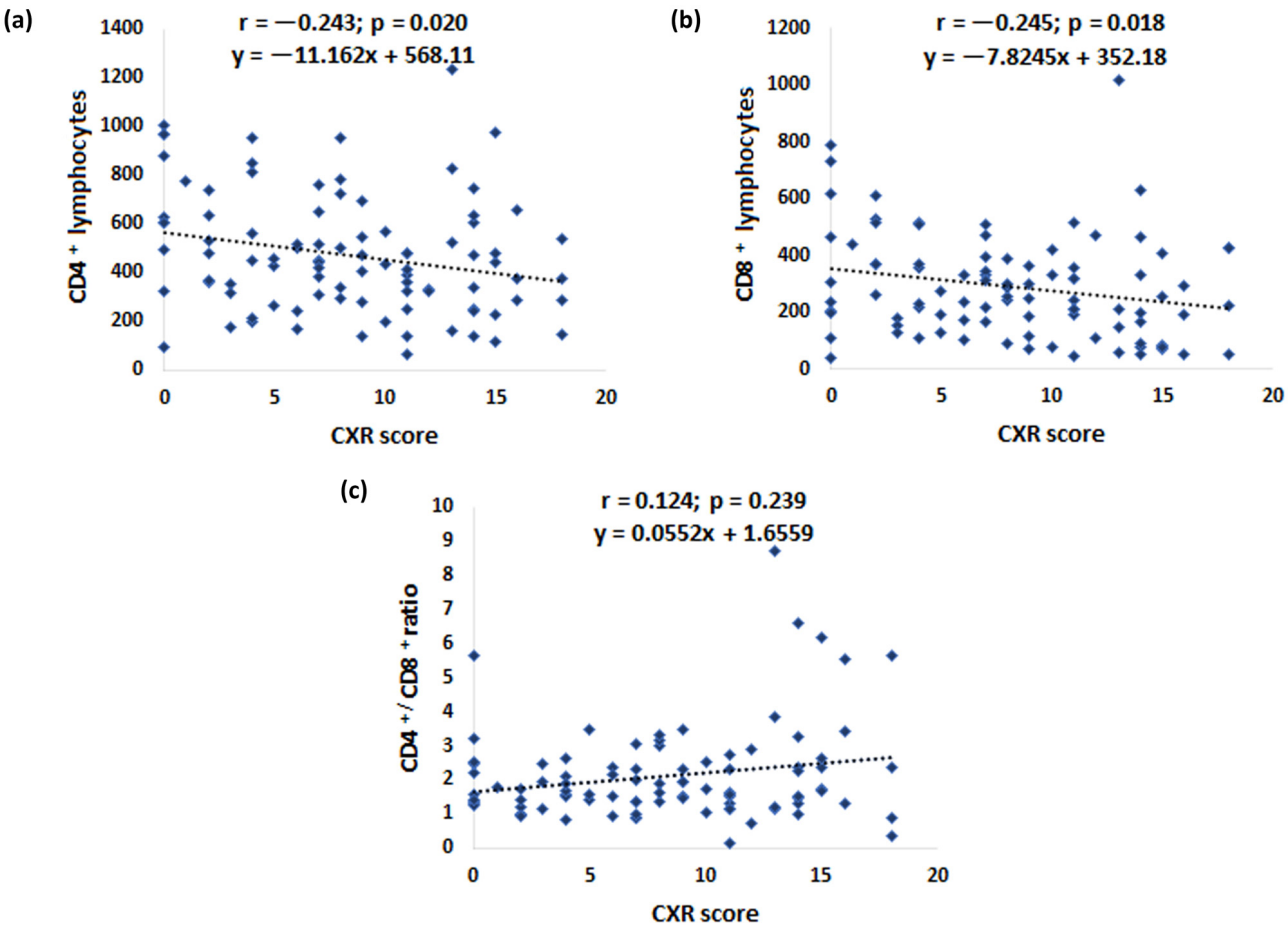
Blood CD4+, CD8+ lymphocyte values and CD4+/CD8+ ratio are related to CXR score in COVID-19-positive patients. Data were obtained from the medical history and blood samples were taken straight after hospital admission. X-ray images of the chest were separated into six zones by two lines. The sum of the scores of each zone (range: 0–18) was calculated based on the lung changes and presented as CXR score. Corresponding to this score, the findings were categorized as normal, interstitial infiltrates, focal, or multifocal consolidations (*n* = 101). (a) Correlations of blood CD4+ lymphocyte values, (b) CD8 lymphocyte values, and (c) CD4+/CD8+ ratio values with the lung changes determined by the CXR score. Spearman’s correlation coefficient was used to determine statistical significance. Values of *p* < 0.05 were taken as statistically significant.

We used radiographic score to assess the severity of the clinical presentation. An escalation in this score correlated with a reduction of CD4+ and CD8+ lymphocyte values in blood (Figure 2a and b). Furthermore, there was a slight simultaneous increase in the CD4+/CD8+ ratio (Figure 2c).

### 3.3 An increase in the values of CD4+ lymphocytes corresponds to a rise in CD8+ lymphocyte values, whereas an increase in CD8+ lymphocyte values leads to a decrease in the CD4+/CD8+ ratio

**Figure 3:**
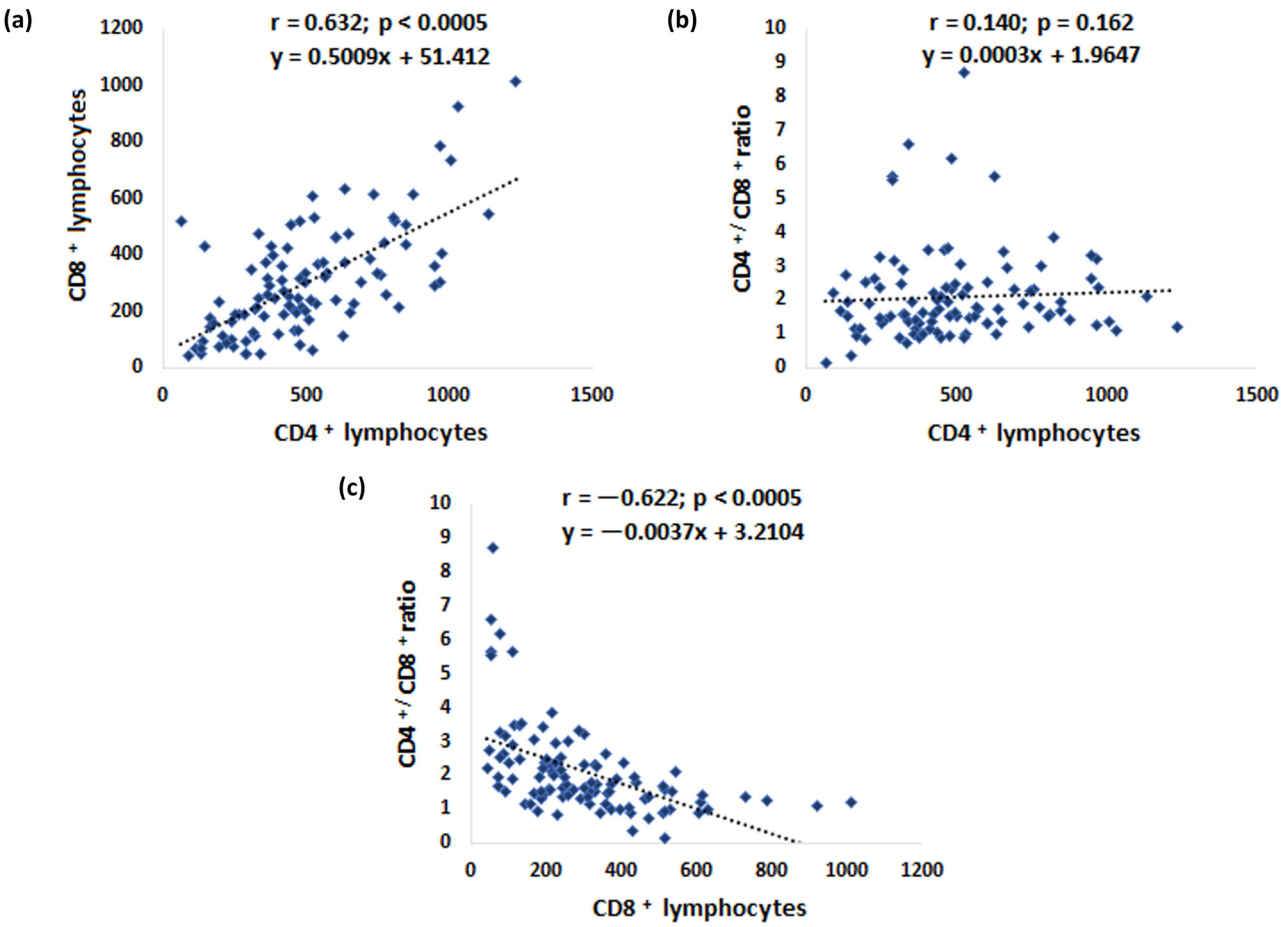
Correlations between CD4+, CD8+ lymphocyte values, and CD4+/CD+ 8 ratio. Data were obtained from the medical history and blood samples were taken straight after hospital admission. (a) Correlations of blood CD4+ and CD8+ lymphocyte values, (b) CD4 lymphocyte values and CD4+/CD8+ ratio, and (c) CD8+ lymphocyte values and CD4+/CD8+ ratio. Spearman’s correlation coefficient was used to determine statistical significance. Values of *p* < 0.05 were taken as statistically significant.

It was observed that patients with higher CD4+ lymphocyte values also had higher CD8+ lymphocyte values (Figure 3a). However, with an increase in the number of CD8+ lymphocytes, there was a significant decrease in the CD4+/CD8+ lymphocyte ratio (Figure 3c), in contrast to an increase in CD4+ lymphocytes (Figure 3b).

### 3.4 Blood values of CD4+ and CD8+ lymphocytes proved to be significant factors for predicting the severity of the clinical picture, the outcome of the disease, and the need for oxygen therapy

**Figure 4:**
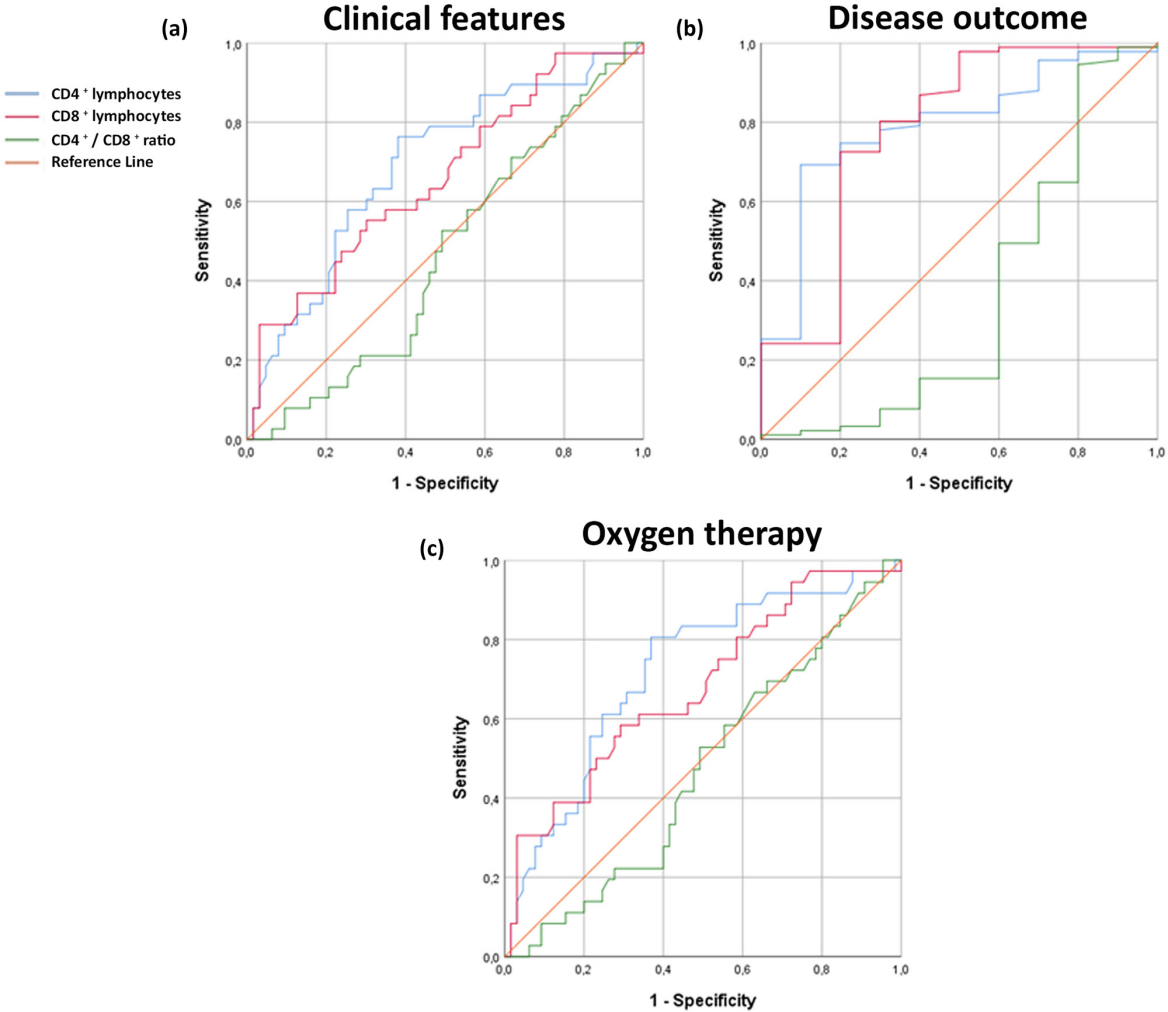
ROC analysis of CD4+ and CD8+ lymphocyte values, as well as CD4+/CD8+ ratio as predicting variables for the severity of the clinical picture, the outcome of the disease, and need for oxygen therapy. According to the disease severity the patients were divided into groups with mild (*n* = 38) and severe (*n* = 63) while according to the disease outcome into groups with favorable (*n* = 91) and fatal (*n* = 10) outcome. Considering the need for oxygen therapy, the patients were divided into a group that was treated with oxygen (*n* = 65) and the other that was not (*n* = 36). Data were obtained from the medical history and blood samples were taken straight after hospital admission. (a) Blood CD4+ and CD8+ lymphocyte values point to the severe clinical presentation of the disease. (b) Blood CD4+ and CD8+ lymphocyte values point to the development of a fatal outcome. (c) Values of blood CD4+ and CD8+ lymphocytes point to the need for oxygen therapy. Statistical significance was determined using the ROC curve. Values of *p* < 0.05 were taken as statistically significant.

In patients with COVID-19, blood CD4+ lymphocyte values below the cut-off value of 472.5 cells/mm^3^ may indicate a severe clinical presentation (AUROC: 0.692, sensitivity: 68.4%, specificity: 63.5%, *p* = 0.001). Values lower than 259 cells/mm^3^ may indicate a higher risk of a fatal disease outcome (AUROC: 0.720, sensitivity: 72.2%, specificity: 64.6%, *p* = 0.000), and values lower than 324.5 cells/mm^3^ may suggest a need for oxygen therapy (AUROC: 0.791, sensitivity: 78%, specificity: 70%, *p* = 0.003) (Figure 4a–c).

Additionally, blood CD8+ lymphocyte values below 259 cells/mm^3^ are characteristic of patients with a severe clinical presentation (AUROC: 0.660, sensitivity: 60.5%, specificity: 39.5%, *p* = 0.007). Values lower than 281 cells/mm^3^ may point at a lethal outcome (AUROC: 0.678, sensitivity: 61.1%, specificity: 60%, *p* = 0.003), and values lower than 167 cells/mm^3^ indicate a higher risk of a lethal outcome (AUROC: 0.782, sensitivity: 80.2%, specificity: 70%, *p* = 0.004) (Figure 4a–c). The CD4+/CD8+ ratio did not demonstrate strong predictive performance as a parameter for assessing the severity of the clinical presentation, disease outcome, and the requirement for oxygen therapy.

### 3.5 Relationship of blood CD4+ and CD8+ lymphocyte values and CD4+/CD8+ ratio in COVID-19 patients

The differences between the values of CD4+ and CD8+ lymphocytes in blood as well as CD4+/CD8+ ratio depending on sociodemographic characteristics and comorbidities are shown in Table 2.

**Table 2:** Correlations of CD4+ and CD8+ lymphocyte values, as well as CD4+/CD8+ ratio with sociodemographic characteristics and comorbidities in COVID-19-positive patients (*n* = 101)

		*N* (%)	CD4 lymphocytes		CD8 lymphocytes		CD4/CD8 ratio	
Variables			M (1Q, 3Q)	*p* Value	M (1Q, 3Q)	*p* Value	M (1Q, 3Q)	*p* Value
Gender	Males	68 (67.3%)	430.5 (283.8, 557.3)	0.032	251 (147.8, 368.8)	0.221	1.6 (1.2, 2.4)	0.317
	Females	33 (32.7%)	566 (351, 750)		317 (180, 462)		1.8 (1.4, 2.5)	
Comorbidities	No	41 (40.6%)	481 (337, 681.5)	0.342	302 (168, 437.5)	0.426	1.7 (1.3, 2.1)	0.774
	Yes	60 (59.4%)	453 (297, 621.3)		244 (166.5, 394.3)		1.7 (1.3, 2.6)	
Diabetes mellitus	No	84 (83.2%)	448.5 (297, 621.3)	0.217	257.5 (169.8, 425.5)	0.611	1.6 (1.2, 2.4)	0.052
	Yes	17 (16.8%)	523 (369.5, 751.5)		258 (124.5, 359.5)		2.2 (1.6, 3.4)	
Arterial hypertension	No	54 (53.5%)	506.5 (357.3, 705)	0.058	306 (195.8, 445.5)	0.095	1.7 (1.3, 2.3)	0.796
	Yes	47 (46.5%)	405 (282, 602)		232 (133, 360)		1.7 (1.3, 2.8)	

Data were obtained from the medical history and blood samples were taken straight after hospital admission. The Mann–Whitney *U*-test was used to determine statistical significance. Results were depicted as median (1Q, 3Q). Values of *p* < 0.05 were taken as statistically significant. M – median.

### 3.6 Influence of lymphocytes, CRP, ferritin, and iron values on the blood CD4+ and CD8+ values

We have performed multiple linear logistic regression to examine the prediction of CD4+ lymphocyte values. The model consisted of four independent variables (lymphocytes [Ly], CRP, ferritin, and serum iron [Fe]) that had been selected according to the initial univariate analysis concerning multicollinearity principles.

Model was statistically significant for the prediction of CD4+ lymphocyte values (*R* ^2^ = 0.52, *F* (4, 73) = 19.625, *p* = < 0.0005). The fitted regression model was *y* = 270.950 + 247.260 × (Ly) – 0.366 × (CRP) − 0.073 × (ferritin) + 5.861 × (Fe). It was found that only one independent variable (Ly) gave statistically significant contribution to the model (*β* = 5.861, *p* = <0.0005) (Table 3).

**Table 3:** Multiple linear logistic regression model consisting of four independent variables (Ly, CRP, ferritin, Fe)

	Unstandardized coefficients		Standardized coefficients			95.0% Confidence interval for *B*	
	*B*	Std. error	Beta	*t*	Sig.	Lower bound	Upper bound
(Constant)	270.950	65.484		4.138	0.000	140.440	401.460
Ly	247.260	41.352	0.552	5.979	0.000	164.846	329.673
CRP	−0.366	0.413	−0.099	−0.885	0.379	−1.189	0.457
Feritin	−0.073	0.053	−0.150	−1.376	0.173	−0.180	0.033
Fe	5.861	4.985	0.106	1.176	0.244	−4.075	15.797

An identical model was used to examine the prediction of CD8+ lymphocyte values (Table 4). In this model, we examined the other four independent variables (Ly, CRP, gamma-glutamyl transferase [gGT], and ferritin). The model was statistically significant for the prediction of CD8+ lymphocyte values (*R* ^2^ = 0.401, *F* (4, 70) = 11.712, *p* = <0.0005). The fitted regression model was *y* = 156.042 + 190.816 × (Ly) + 0.311 × (CRP) − 0.538 × (gGT) – 0.070 × (ferritin). Only one independent variable (lymphocytes) gave statistically significant contribution to the model (*β* = 190.816, *p* = < 0.0005).

**Table 4:** Multiple linear logistic regression model consisting of four independent variables (lymphocytes, CRP, gGT, ferritin)

	Unstandardized coefficients		Standardized coefficients			95.0% confidence interval for *B*	
	*B*	Std. error	Beta	*t*	Sig.	Lower bound	Upper bound
(Constant)	156.042	52.426		2.976	0.004	51.482	260.601
Ly	190.816	32.753	0.580	5.826	0.000	125.492	256.140
CRP	0.311	0.321	0.112	0.968	0.336	−0.330	0.951
gGT	−0.538	0.487	−0.106	−1.105	0.273	−1.510	0.433
Feritin	−0.070	0.044	−0.181	−1.570	0.121	−0.158	0.019

**Figure 5:**
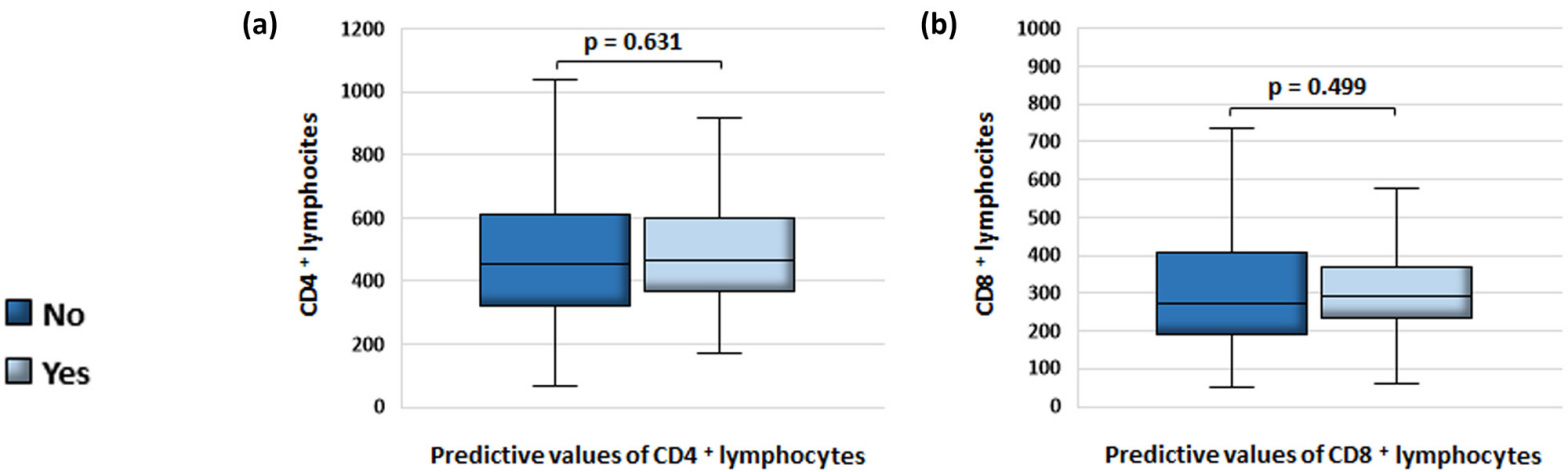
Comparison of CD4+ and CD8+ lymphocytes from blood samples with the values predicted by the equation obtained by the multiple linear regression model. (a) There is no difference between the experimentally obtained and the predicted values of CD4+ (Mdn = 453.50 cells/mm^3^ (322, 609.75) vs Mdn = 467.53 cells/mm^3^ (368.58, 597.21), *n* = 78; *p* = 0.631) and (b) CD8+ lymphocytes (Mdn = 274 cells/mm^3^ (189, 407) vs Mdn = 293.85 cells/mm^3^ (231.87, 369.13), *n* = 75; *p* = 0.499). The Wilcoxon signed-rank test was used to determine statistical significance. Results were depicted as median (1Q, 3Q). Values of *p* < 0.05 were taken as statistically significant.

Finally, we tested the regression equations and determined that there is no difference between the predicted and experimentally obtained values of CD4+ and CD8+ lymphocytes (Figure 5).

## 4 Discussion

In late 2019, a new strain of β-coronavirus was discovered, which was named severe acute respiratory syndrome coronavirus 2 [19]. The intensive spread of the disease was accompanied by a significant number of patients with a severe clinical picture characterized by acute respiratory distress syndrome, coagulation disorders, and systemic disorders. This situation emphasized the need for rapid identification of epidemiological, diagnostic, and other relevant parameters that could be used to assess and predict the development of the clinical picture of patients in the early stages of treatment. Therefore, the main goal of this study was to evaluate the predictive significance of certain risk factors, primarily chronic diseases, and basic laboratory and radiographic analyses, on the development of a severe clinical picture and death in patients with COVID-19.

The analysis of demographic characteristics showed that gender differences could significantly affect the prognosis of the disease and the need to use oxygen therapy. As shown in Table 1, a higher percentage of male patients had a severe form of the disease and the need for oxygen therapy compared to female patients. These results follow many studies which, since the beginning of the pandemic, have shown that there is a significantly higher risk of severe forms of the disease as well as higher mortality in men [20,21]. In most studies in which patients from different countries were examined, a male bias in COVID-19 mortality was shown [22].

A similar discrepancy had previously been observed during the severe acute respiratory syndrome and Middle Eastern respiratory syndrome coronavirus epidemics that broke out in previous decades (caused by SARS-CoV and MERS-CoV, respectively) [20,23].

Chromosomal variations that are the basis of the biological differences between the sexes cause hormonal differences that are important in the regulation of metabolism and affect the degree of risk of various diseases. The protective role of estrogen has been demonstrated in metabolic and cardiovascular diseases [24–26]. We have shown that the presence of comorbidities, arterial hypertension, and diabetes mellitus in COVID-19 patients is associated with the development of a more severe disease, which is in accordance with previous studies [27]. Differences between the sexes, which can be significant in morbidity, are also reflected in differences in life habits such as lifestyle and social habits, and consumption of tobacco and alcohol [7].

Biological gender differences also cause differences in the immune response, both innate and adaptive, as well as the response to infections [22,28,29]. Known sex differences that may impact immune responses to SARS-CoV-2 and COVID-19 progression involve molecular mechanisms that are determined to a large extent by genes on sex chromosomes. Angiotensin-converting enzyme 2 (ACE2) is an entry receptor used by SARS-CoV-2 [30]. ACE2 is an X chromosome-encoded gene, downregulated by estrogens and exhibits tissue-specific expression patterns [31,32]. Virus entry enhanced by cellular transmembrane serine protease 2 (TMPRSS2), which primes the spike protein of the virus [30] and the expression of TMPRSS2 is regulated by androgens [33]. Considering the different gender expression and regulation of these two molecules, this mechanism may be relevant for the different disease susceptibility in men, but further research is needed to confirm this theory. In the early stages of the immune response to viruses, innate sensing and interferon production play a significant role [34]. Toll-like receptor 7, a pattern-recognition receptor, has the key role in the innate sensing of viral RNA. It has been shown that this molecule escapes X chromosome inactivation, which results in its greater expression on immune cells in women [35]. It has also been shown that certain subsets of innate cells produce cytokines differently depending on gender.

Production of interferon-α in plasmacytoid dendritic cells, dependent on sex hormones, has already been shown, which may also be a potential mechanism relevant to the development of the disease [36,37].

The immune response to viral infections is characterized by the activation of mechanisms of innate and acquired immunity. Activation and effector mechanisms of cellular immunity, i.e., T lymphocytes, are particularly important in immune defense against viruses.

Our results showed, as presented in Figure 1, that the number of CD4+ and CD8+ lymphocytes in the peripheral blood was significantly lower in patients who developed a severe form of the disease, in fatal outcomes, as well as in patients whose treatment required oxygen therapy. At the same time, the CD4+/CD8+ ratio was not significantly different in the context of disease severity and mortality. The results of the presented study are following the results of previous studies in which a significant decrease in the number of lymphocytes in the peripheral blood of COVID-19 patients was shown, especially in severe forms of the disease that required treatment in intensive care units, often with a fatal outcome [38–40]. Also, the CD4+/CD8+ ratio was in the normal range in our patients, which is consistent with previous studies [41]. There is a plethora of evidence that SARS-CoV-2 infection leads to a decrease of lymphocyte subsets, but the mechanisms are still unclear. Some evidence indicates that T cell exhaustion occurs during infection, defined as a state of T cell dysfunction that arises during many chronic infections, characterized by poor effector function, sustained expression of inhibitory receptors, and a transcriptional state distinct from that of functional effector or memory T cells [42]. Studies have shown that this phenomenon is associated with changes in serum levels of cytokines such as IL-10, IL-6, and TNF-α [38]. CD4+/CD8+ ratio has been used as a quantitative prognostic risk factor in viral infections and some studies demonstrated its role in COVID-19 prediction of disease severity [43].

Our results showed that, although there was a comparable trend, the values of this prognostic factor did not differ significantly between the studied groups. A possible explanation probably lies in the limited number of the study population. Analysis using the ROC curve showed that the decrease in the number of CD4+ and CD8+ lymphocytes in the peripheral blood has a higher sensitivity and specificity concerning the CD4+/CD8+ ratio, as shown in Figure 4.

Different modalities of lung radiography have proven to be a very useful tool in evaluating the outcome and planning the treatment of COVID-19 patients, considering that evaluation of disease severity based solely on clinical judgment in the case of associated pneumonia is often insufficient. Accordingly, different disease severity scores have been defined for a quick assessment of the patient’s condition [44,45]. Although CT diagnostics showed high specificity and reliability, a high proportion of COVID-19-induced lesions can also be seen using CXR imaging, which is a significantly more accessible and cost-effective method. As shown in Figure 2, our findings showed that there was a significant negative correlation between the number of CD4+ as well as CD8+ lymphocytes in the blood and the value of the CXR score, which may have predictive significance.

Our results showed that the number of CD4+ and CD8+ lymphocytes could have a prognostic significance in determining the severity of the clinical picture and the death outcome. A CD4+ lymphocyte count below 472.5 cells/mm^3^ may indicate a severe clinical picture, while values lower than 259 cells/mm^3^ may indicate a fatal outcome. On the other hand, a CD8+ cell/mm^3^ count below 259 may have a predictive value for death, while the count below 167 cells/mm^3^ indicates a higher risk of death.

The results of other authors have also shown that reduced values of CD4+ and CD8+ can have predictive significance for the fatal outcome [46].

Reduced values of lymphocytes indicate a significant degree of cellular immunodeficiency, which is why the appearance of opportunistic infections in the most severe patients was expected.

Our research also has its limitations. In the first place, it is a small number of samples, and in the second place, we did not monitor the development of the cellular immune response in different stages of the disease, but only the initially taken values of these parameters.

Future studies should aim to investigate the benefits of measuring lymphocyte subtype levels as prognostic biomarkers for disease severity, mortality, and response to treatment in patients infected with the SARS-CoV-2 virus. A comprehensive understanding of these immune mechanisms is crucial for devising effective strategies to manage and prevent COVID-19, thereby enhancing patient outcomes and mitigating the pandemic’s impact.

## 5 Conclusion

The results of this study demonstrated the predictive significance of CD4+ and CD8+ peripheral blood cells in COVID-19 patients. Reduced values of CD4+ and CD8+ correlate with a severe clinical picture, which is followed by a high CXR score and the fatal outcome of the disease. In this study, we found gender-dependent differences, but future studies should be aimed at identifying and examining factors that lead to differences in the immune response depending on gender. Since these findings on our patient population are in accordance with similar findings by other authors, we might consider these laboratory parameters as relevant and useful markers in the prognosis of COVID-19.

## Abbreviations

ACE2: angiotensin-converting enzyme 2
AUROC: area under the receiver operating characteristic curve
BMI: body mass index
CXR: chest X-rays
CRP: C-reactive protein
Fe: serum iron
gGT: gamma-glutamyl transferase
Ly: lymphocytes
Mdn: median
ROC: receiver operating characteristic
TMPRSS2: cellular transmembrane serine protease 2
